# Association of fibronectin 1 deregulation with tyrosine kinase inhibitor resistance in chronic myeloid leukemia

**DOI:** 10.3389/fcell.2025.1725857

**Published:** 2025-12-19

**Authors:** Lina Tiedemann, Sivahari Prasad Gorantla, Philine Ahlf, Lucie Sophie Schmidt, Christiane Pott, Merit Litterst, Vicki Waetzig, Inga Nagel, Johanna Ruemenapp, Nikolas von Bubnoff, Ingolf Cascorbi, Meike Kaehler

**Affiliations:** 1 Institute of Experimental and Clinical Pharmacology, University Hospital Schleswig-Holstein, Campus Kiel, Kiel, Germany; 2 Department of Hematology and Oncology, University Hospital Schleswig-Holstein, Campus Lübeck, Lübeck, Germany; 3 Second Medical Department, University Hospital Schleswig-Holstein, Campus Kiel, Kiel, Germany; 4 Institute of Human Genetics, University Hospital Schleswig-Holstein, Campus Kiel, Kiel, Germany; 5 Division of Neurological Pain Research and Therapy, Clinic for Neurology, University Hospital Schleswig-Holstein, Campus Kiel, Kiel, Germany

**Keywords:** chronic myeloid leukemia, drug resistance, fibronectin 1, imatinib, tyrosine kinase inhibitor

## Abstract

**Introduction:**

Therapy of chronic myeloid leukemia (CML) with tyrosine kinase inhibitors (TKIs) targeting the *BCR::ABL1* kinase has become a paradigm for precision oncology. Despite the tremendous success of this strategy, with an overall long-term survival rate of 83%, approximately 25% of CML patients experience therapy failure within 5 years of treatment. TKI resistance is multifaceted, involving mutations in *BCR::ABL1*, but also BCR::ABL1-independent mechanisms. Among them, deregulation of cell adhesion and motility of CML cells has been observed in TKI-resistance. The extracellular matrix protein fibronectin 1 (FN1) has been shown to be deregulated in solid tumors promoting proliferation and metastasis. However, the role of FN1 in hematopoietic neoplasms remains to be fully elucidated. The aim of our study was to gain deeper insights into the role of FN1.

**Methods:**

*FN1* mRNA and protein levels were analyzed using qPCR and immunoblotting. Transfection was performed using nucleofection or stable transfection, followed by analyses of cell number, proliferation and viability. Cell adhesion was assessed using Matrigel-coated surfaces, and FN1 localization was analyzed using immunofluorescence.

**Results:**

FN1 levels were significantly downregulated in CML cell lines resistant against BCR::ABL1 inhibitors *in vitro*. SiRNA-mediated *FN1* knockdown reduced the cell’s susceptibility to all generations of TKIs employed in treatment of CML, including asciminib. In contrast, the restoration of *FN1* expression in TKI-resistant cells re-sensitized the cells to TKI treatment. This effect was also observed in K-562 cells that intrinsically harbor the *BCR::ABL1* mutation p. E255K (−35.2%, *p* < 0.001), as well as in K-562 and Ba/F3 cells after stable transfection of the BCR::ABL1 wild-type or the p. T315I gatekeeper mutation. Clinically, deregulation of *FN1* was also observed in peripheral blood cells derived from CML patients.

**Conclusion:**

Our data indicate that FN1 may serve as a potential therapeutic target to address TKI resistance or as a suitable biomarker for the treatment.

## Graphical Abstract

**Figure:**
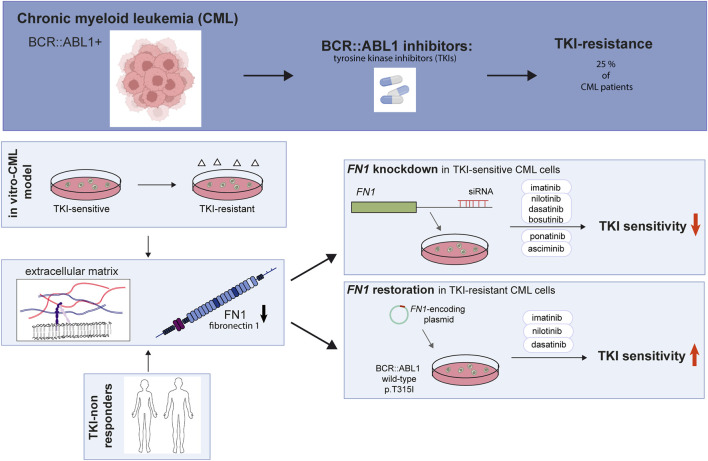

## 1 Introduction

Targeted therapies that address the fundamental mechanisms of tumor development are increasingly used in hemato-oncology. The paradigm disease for such treatments has been chronic myeloid leukemia (CML), where understanding of the disease mechanism led to the development of the first-generation tyrosine kinase inhibitor imatinib (Buchdunger et al., 1996; Druker et al., 1996). Targeting the disease-causing oncoprotein, the BCR::ABL1 kinase, has resulted in tremendous therapeutic success. The *BCR::ABL1* fusion gene typically arises from the reciprocal translocation t (9; 22) (q34; q11) and is the predominant cause of this rare myeloproliferative neoplasm (Deininger et al., 2000).

Since the development of tyrosine kinase inhibitors (TKIs), which bind to the BCR::ABL1 kinase and thereby inhibit downstream target phosphorylation, CML can be effectively treated resulting in overall 10-year survival rates reaching 83% (Hochhaus et al., 2017). However, up to 25% of CML patients require alternative TKI therapies within 5 years of treatment initiation (Milojkovic and Apperley, 2009). Consequently, subsequent generations of TKIs - namely, nilotinib, dasatinib, bosutinib, ponatinib, and the STAMP (specifically targeting the Abl myristoyl pocket)-inhibitor asciminib - have been designed to overcome resistance. These TKIs vary in potency, specificity, and adverse effects and can be used with variable success in the treatment of imatinib-resistant patients (Hochhaus et al., 2020; Perez-Lamas et al., 2023).

TKI resistance arises from BCR::ABL1-dependent or -independent mechanisms (Bixby and Talpaz, 2011). In approximately half of the cases, mutations of *BCR::ABL1,* as well as its overexpression or amplification, can be observed. Mutations such as p. Tyr253His (Y253H), p. Glu255Val (E255V) or the gatekeeper mutation p. Tyr315Ile (T315I) impair TKI binding to the BCR::ABL1 ATP-binding pocket, resulting in the loss of previously achieved remission (O'Hare et al., 2007; Zabriskie et al., 2014). BCR::ABL1-independent resistance involves various proposed mechanisms, including deregulation of drug transporter expression, alterations in gene expression, activation of alternative signaling pathways, and the persistence of leukemic stem cells (Tauchi and Ohyashiki, 2004; Kaehler and Cascorbi, 2021).

In CML, studies have demonstrated sustained cell motility and decreased cell adhesion, both of which were shown to be reversed during TKI treatment (Chen et al., 2013). Furthermore, deregulated cell adhesion properties have been associated with TKI resistance (Kumar et al., 2020). Fibronectin 1 (FN1) is a 200–250 kDa heterodimeric protein located in the extracellular matrix (ECM) consisting of 47 exons and expressed in 20 isoforms (Pankov and Yamada, 2002). FN1 is produced either by hepatocytes as soluble plasma fibronectin, essential for blood coagulation, or as insoluble, cellular fibronectin, synthesized by epithelial cells, fibroblasts, and macrophages, where its functions as a key component of cellular matrix protein (To and Midwood, 2011; Spada et al., 2021). Known to be involved in cell adhesion signaling via integrins, focal adhesion kinase and the integrin-like kinase (Kumar et al., 2017), FN1 plays a role in cell differentiation and metastasis formation. Its deregulation has been observed in several solid tumors, including lung, gallbladder, and ovarian cancer (Meng et al., 2009; Yousif, 2014; Cao et al., 2015; Lin et al., 2019). However, studies on its role in hematopoietic tumors, including CML, remain limited.

In a previous study, we demonstrated that *FN1* is downregulated in an *in vitro* model of TKI resistant CML. Furthermore, we showed that *FN1* knockdown promotes imatinib resistance, while restoration of *FN1* expression in imatinib-resistant cells leads to reestablishment of TKI susceptibility (Kaehler et al., 2022). However, the study was limited to imatinib-resistant K-562 cells. The present study investigates whether *FN1* deregulation is a recurrent phenomenon in TKI-resistant leukemia. To validate our previous findings in K-562 cells, we extended our analysis to LAMA-84 and NALM-20 leukemic cell lines, which are both TKI-resistant and BCR::ABL1-positive. We also examined whether *FN1* regulation is dependent on the presence of *BCR::ABL1* mutations. In addition, we explored the underlying mechanisms contributing to FN1-associated TKI resistance. Finally, to assess the *in vivo* relevance of *FN1* in TKI resistance, *FN1* expression was quantified in peripheral blood cells from CML patients at diagnosis and during relapse under TKI therapy.

## 2 Materials and methods

### 2.1 Reagents and cell lines

If not indicated otherwise, reagents were obtained from Sigma-Aldrich (Darmstadt, Germany) or Carl Roth (Karlsruhe, Germany).

K-562 cells (RRID: CVCL_0004), LAMA-84 (RRID: CVCL_0388), NALM-20 cells (RRID: CVCL_1836) and Ba/F3 pro-B cells (RRID: CVCL_0161) were obtained from the German Collection of Microorganisms and Cell Cultures (DSMZ, Braunschweig, Germany) and cultivated as described elsewhere (Gorantla et al., 2010; Turrini et al., 2012; Kaehler et al., 2017). NALM-20 cells were maintained in RPMI-1640 media (Thermo Fisher Scientific, Darmstadt, Germany) diluted with 20% v/v FBS (Bio & Sell, Feucht, Germany). Cell line authentication was performed via STR profiling by Eurofins Genomics (Ebersberg, Germany). All cells were mycoplasma-free as analyzed with the VenorGeM OneStep Kit (Minerva Biolabs, Berlin, Germany).

### 2.2 Generation of TKI-resistant cells

Generation of biological replicates of TKI-resistant sublines was performed as described elsewhere (Turrini et al., 2012; Kaehler et al., 2017; Kaehler et al., 2022). Briefly, treatment-naïve K-562 cells were exposed to increasing TKI concentrations to obtain four biological replicates of cells resistant to 2 µM imatinib, two replicates of 0.1 µM nilotinib-resistant or two replicates of 0.01 µM dasatinib-resistant cells. Dual imatinib- and nilotinib-resistant cells were generated in two replicates. For this purpose, 2 µM imatinib-resistant cells were stepwise exposed to a final concentration of 0.1 µM nilotinib and *vice versa*. Resistance was likewise established for LAMA-84 cell line resistant to 0.5 and 2 µM imatinib (Kaehler et al., 2021) and NALM-20 cells resistant to 0.1 µM imatinib. Analysis of *BCR::ABL1* mutations was performed as previously described (Kaehler et al., 2017).

### 2.3 Patient samples

Peripheral blood from 13 healthy volunteers (age: 21–57, median: 29 years) was collected and RNA was isolated using the NucleoSpin RNA Blood Kit (Macherey-Nagel, Düren, Germany) according to the manufacturer’s protocol. Blood samples from CML patients derived from initial diagnosis (age: 8–88, median: 66 years) were analyzed as previously described (Kaehler et al., 2021). Left-over RNA extracted from peripheral blood from ten TKI-non responding CML patients (age: 33–93; median: 68 years) for routine treatment monitoring of *BCR::ABL1* fusion transcript under TKI treatment was provided by the Hämatologie Labor Kiel, Second Medical Department, University Hospital Schleswig-Holstein, Kiel, Germany. The presence of *BCR::ABL1* fusion transcripts was confirmed by consensus multiplex PCR and breakpoint determination. In case of poor response to TKI treatment, Sanger sequencing was performed to identify secondary point mutations within the *BCR::ABL1* tyrosine kinase domain. Major molecular response (MMR) to TKI treatment was defined by a *BCR::ABL1* transcript ≤0.1% calibrated to the International Scale (IS) for *BCR::ABL1* measurement. Non-responders were defined as patients, who initially showed a reduction in the *BCR::ABL1* transcript after TKI onset, but then relapsed. Detailed patient data are given in Supplementary Table 1. The procedures were performed according to the ethical standards of the institutional and national research committee (Ethics Committee of the Medical Faculty of Kiel University, D426/03 and D114/05), the declaration of Helsinki and its later amendments or comparable ethical standards. All patients and volunteers gave their written consent.

### 2.4 RNA extraction and reverse transcription-quantitative polymerase chain reaction (RT-qPCR)

Total cell line RNA was isolated using the PeqGOLD TriFast (VWR Life Science, Darmstadt, Germany) or the E. Z.N.A. Total RNA Kit I. (Omega bio-tek, Norcross, GA, USA) according to the manufacturer’s protocol. 1 μg RNA was reversely transcribed using the High-Capacity cDNA Reverse Transcription Kit (Thermo Fisher Scientific) according to the manufacturer’s recommendations. RT-qPCR was performed using TaqMan Universal Master Mix II, with UNG (Thermo Fisher Scientific) and the assays *FN1* (Hs01549976_m1), *SPARC* (Hs00234160_m1), *COL15A1* (Hs00266332_m1), *GAPDH* (Hs02786624_g1), *TBP* (Hs00427620_m1) and *18S* (Hs99999901_s1) with default cycling conditions. Analysis was run on the QuantStudio 7 Flex (Thermo Fisher Scientific). Statistical analysis using the 2^−ΔΔCt^-method was performed as previously described (Kaehler et al., 2021).

### 2.5 Whole cell lysates and immunoblotting

Lysis and immunoblotting were performed as described elsewhere using 1 × 10^6^ cells, denaturating lysis buffer and 20 µg protein/sample (Kaehler et al., 2017; Waetzig et al., 2019; Bruhn et al., 2020). The Plasma Membrane Protein Extraction Kit (Abcam, Cambridge, United Kingdom) was used for enrichment of membrane proteins (Kaehler et al., 2022). Blots were probed with the following antibodies: Fibronectin: Clone P1H11, Cat# MAB 1918, RRID: AB_2105831, 1:500 (R&D Systems, Minneapolis, USA); HSP90: Clone C45G5, Cat# 4877, RRID: AB_2233307, 1:2,000; p-p38: Cat#9211, RRID: AB_331641, 1:500 (both Cell Signaling Technology, Danvers, USA); p-38: sc-7972, RRID: AB_628079, 1:1,000 (Santa Cruz Biotechnology, Dallas, USA); anti-mouse: Cat# 926-32210, RRID: AB_621842 and anti-rabbit: Cat# 926-68071, RRID: AB_10956166; all 1:10,000 (LI-COR Biosciences, Bad Homburg, Germany). Primary antibodies were diluted in Intercept/TBS blocking solution (LI-COR) supplemented with 0.2% v/v Tween-20, secondary antibodies in TBS with 0.1% v/v Tween-20.

### 2.6 Immunofluorescence

1 × 10^6^ cells/mL were seeded onto sterile coverslips coated with 0.01% w/v poly-l-lysine in a 6 well-plate and incubated for 24 h at 37 °C. The cells were washed with ice-cold PBS and fixed with 4% w/v paraformaldehyde/PBS pH 7.4 (Thermo Fisher Scientific) for 10 min, followed by three washing steps with PBS. 0.5% w/v saponin/PBS was added for 10 min to permeabilize the cells including washing for three times with PBS for 5 min. The coverslips were transferred into new six well-plates and 1% w/v BSA/PBS plus 0.1% v/v Tween-20 (PBST) was added. After 30 min, the cells were probed with the following primary antibodies diluted in 1% w/v BSA/PBST for 1 h at room temperature: Fibronectin: Clone P1H11, Cat# MAB 1918, RRID: AB_2105831, 1:50 (R&D Systems); LAMP1: Cat# ab24170, RRID: AB_775978, 1:100 (Abcam); Vimentin: Clone SP20, Cat# MA5-16409, RRID: AB_2537928, 1:1,000; ZO-1: Cat# 61-7,300, RRID: AB_2533938, 1:25 (both Thermo Fisher Scientific). Coverslips were washed three times with PBS for 5 min before adding the secondary antibodies (Thermo Fisher Scientific) for 1 h in the dark: anti-mouse Alexa Fluor 488: Cat# R37114, RRID: AB_2556542; anti-rabbit Alexa Fluor 594: Cat# R37117, RRID: AB_2556545; diluted according to the manufacturer’s recommendations. After washing three times with PBS for 5 min, the coverslips were embedded onto microscope slides with Prolong Diamond Antifade Mountant with DAPI (Thermo Fisher Scientific). Microscopy was performed using an Axiovert 200M microscope with Plan-Apochromat 63x/140 Oil M27 object glass (Carl Zeiss, Oberkochen, Germany). Quantification was performed using ImageJ2 (http://imagej.nih.gov/ij (Rasband, 1997)).

### 2.7 Cell adhesion assay

Dark 96 well-plates were coated with Corning Matrigel Basement Membrane Matrix and adhesion was analyzed using the Vybrant Cell Adhesion Assay (Thermo Fisher Scientific) and 1.5 × 10^6^ cells/sample after 30 min binding to the coated surface as previously described (Kaehler et al., 2022). Fluorescent cells binding to Matrigel were measured at an Infinite M200 Pro device (Tecan Group, Crailsheim, Germany) with 494 nm absorbance and 517 nm emission.

### 2.8 Transient transfection

Transient transfection was either performed using the nucleofector 2b device and the Amaxa Cell Line Nucleofector Kit V or the nucleofector 4D device and the SF Cell Line 4D Nucleofector Kit X (all Lonza, Basel, Switzerland). 3–5 × 10^6^ K-562 or Ba/F3 cells were transfected with 5 or 2 µg of the respective plasmid (pSELECT-FN1) or empty vector control (InvivoGen, Toulouse, France). The *FN1* encoding plasmid was cloned as previously described (Kaehler et al., 2022). *SPARC* (NM_003118.4) was amplified using the PrimeSTAR Max Premix (Takara, Saint-Germain-en-Laye, France), an annealing temperature of 58 °C, the primers 5′-AGA​TCA​CCG​GCG​TGT​CGA​CGA​TGA​GGG​CCT​GGA​TCT​TC-3′ and 5′-ATC​TTA​TCA​TGT​CTG​GCC​AGT​TAG​ATC​ACA​AGA​TCC​TTG​TCG-3′ and cloned into pSELECT (Sigma-Aldrich) using the NEBuilder HiFi DNA Assembly Kit and the restriction enzymes BamHI-HF and NheI-HF (both New England Biolabs (NEB), Ipswich, USA). After plasmid transfection, cells were seeded after 1 h onto respective cell culture plates and treated with 2 µM imatinib, 0.1 µM nilotinib, 0.01 µM dasatinib, 0.006 µM ponatinib or 0.05 µM asciminib. For siRNA-mediated knockdown, K-562 cells were transfected with 10 µM Ambion Silencer Pre-designed siRNA 10826 or Pre-miR negative control #1 (Thermo Fisher Scientific). Twenty-four hours after transfection, cells were seeded onto respective cell culture plates followed by exposure to the respective TKI including 0.02 µM bosutinib. The TKI concentrations were chosen to reflect the desired plasma concentrations (Picard et al., 2007; Baccarani et al., 2013) and respective IC50 values, as tested dose-response curves (data not shown). For expression analyses, cells were used 48 h after transfection.

### 2.9 Stable transfection

K-562 and Ba/F3 cells were engineered to express ecotropic murine leukemia virus receptor (EcoR) prior to transduction with the retroviral vector (pMIG) carrying *BCR::ABL1* wild-type or p. T315I mutation as previously described (Yu et al., 2020). Briefly, Phoenix E cells were transiently transfected using Lipofectamine 2000 (Thermo Fisher Scientific) and retroviral stocks were collected twice at 12 h-intervals beginning 24 h after transfection. Cells expressing EcoR were infected by spin infection (1,200 × g, 32 °C, 90 min) using retroviral supernatant supplemented with 4 μg/mL polybrene. After transduction, cells were checked for eGFP expression by FACS.

### 2.10 Cellular fitness assays

Cell numbers were determined using trypan blue staining or Via2-cassettes and the Nucleocounter NC-202 device (Chemometec, Allerod, Denmark). Cell viability was investigated using WST-1 (Sigma-Aldrich) 48 h after incubation with the respective TKI. Proliferation was analyzed using MKI67 ELISA Kit (MyBioSource, San Diego, USA) 24 h after incubation with the respective TKI using 25 µg protein/sample as previously described (Kaehler et al., 2017; Kaehler et al., 2021; Kaehler et al., 2022). Data were analyzed normalizing TKI-treated to non-treated samples.

### 2.11 Genome-wide expression data

Genome-wide gene expression data were derived from the GEO datasets GSE227347 and GSE203342 obtained from HuGene 2.0 ST and Clariom S arrays (both Affymetrix/Thermo Fisher Scientific) comparing four imatinib- and two nilotinib-resistant sublines with treatment-naïve K-562 cells as previously published (Kaehler et al., 2022; Kaehler et al., 2023). STRING interaction networks were obtained using the STRING database (string-db.org. Version 12.0 with medium confidence) for differentially expressed genes (fold change ±2, false discovery rate (FDR)-corrected *p*-value *p* < 0.05) comparing treatment-naïve with imatinib- and nilotinib-resistant sublines. The imatinib-resistant sublines contained three without *BCR::ABL1* mutations and one harboring the *BCR::ABL1* mutation p. E255K.

### 2.12 Software & statistics

Statistical analysis was performed using One-way ANOVA with subsequent Dunnett’s test, unpaired Student’s t-test or Mann-Whitney *U* tests using the GraphPad prism 10 software (GraphPad, San Diego, USA). *N*-numbers indicate the number of independent technical replicates.

## 3 Results

### 3.1 *FN1* is recurrently downregulated in TKI resistance

**FIGURE 1:**
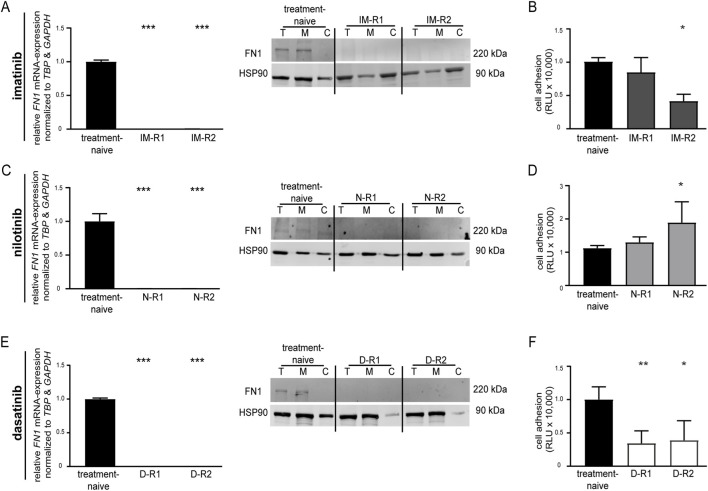
*FN1* expression and cell adhesion properties of TKI-resistant cell lines. *FN1* expression and cell adhesion of (A + B) imatinib- (C + D) nilotinib- and (E + F) dasatinib-resistant cell lines compared to treatment-naïve K-562 cells. **(A,C,E)** *FN1* mRNA expression was measured by RT-qPCR and normalized to *TBP* and *GAPDH* and treatment-naïve cells. Protein levels were obtained by immunoblotting compared to HSP90. **(B,D,F)** Cell adhesion was measured by binding to Matrigel-coated surfaces normalized to treatment-naïve K-562 cells. Data are shown for two biological replicate cell lines (imatinib: IM-R1, IM-R2; nilotinib: N-R1, N-R2; dasatinib: D-R1, D-R2). *N* = 3. Statistical analysis was performed using one-way ANOVA followed by Dunnett’s test. Error bars indicate standard deviation. *p < 0.05, **p < 0.01, ***p < 0.001. T, total protein; M, membrane fraction; C, cytosolic fraction; kDa, kilodalton.

First, FN1 expression was analyzed in two independent biological replicates of K-562 cells that were resistant against imatinib-, nilotinib-, or dasatinib. These cells did not harbor any mutations in BCR::ABL1. In imatinib-resistant cells, *FN1* was significantly downregulated at both the mRNA (IM-R1: *p* < 0.001; IM-R2: *p* < 0.001) and protein levels compared to treatment-naïve cells (Figures 1A,B). Cell adhesion to Matrigel-coated surfaces was significantly decreased in one replicate of imatinib-resistant K-562; however, this effect was not consistent across all imatinib-resistant sublines when compared to treatment-naïve cells (IM-R2: −60.0%, *p* = 0.01, Figure 1C). In LAMA-84 CML cells resistant to 0.5 or 2 µM imatinib, *FN1* mRNA expression was not significantly deregulated, whereas in NALM-20 Ph + ALL cells, *FN1* was undetectable (Supplementary Figure S1).

K-562 cells resistant to the BCR::ABL1 TKIs nilotinib and dasatinib exhibited significantly reduced *FN1* expression compared to treatment-naïve cells at both the mRNA (N-R1: *p* < 0.001, N-R2: *p* < 0.001; D-R1: *p* < 0.001, D-R2: *p* < 0.001) and protein levels (Figures 1A,B). In addition, binding to Matrigel was unaltered in nilotinib resistance, but significantly reduced in dasatinib-resistant cells compared to treatment-naïve controls (D-R1: −65.5%, *p* = 0.006; D-R2: −61.0%, *p* = 0.03, Figures 1C,E; Supplementary Table 2). Downregulated *FN1* expression was also observed in cross-resistant cells derived from sequential exposure to imatinib and nilotinib, whereas cell adhesion was increased (Supplementary Figures S2A,B).

### 3.2 *FN1* knockdown promotes resistance against all generations of BCR::ABL1 TKIs

**FIGURE 2:**
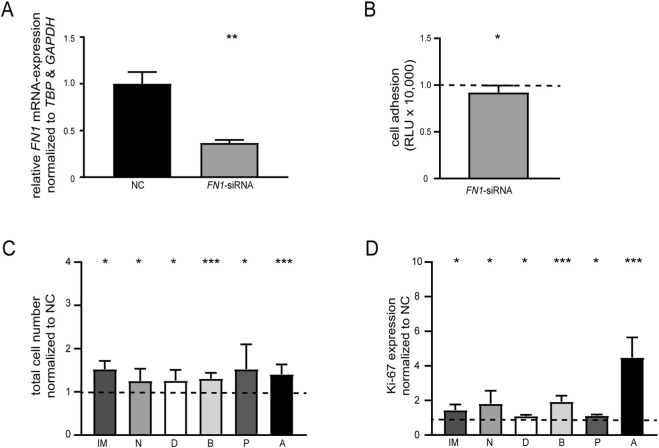
*FN1* knockdown in treatment-naïve K-562 cells decreases susceptibility to TKI treatment. **(A)** *FN1* mRNA expression after *FN1* knockdown by transfection of a *FN1*-specific siRNA into treatment-naïve K-562 cells measured by RT-qPCR and compared to *TBP*, *GAPDH* and negative control-transfected cells (NC). **(B)** Cell adhesion to Matrigel-coated surfaces after *FN1* knockdown. **(C,D)** Cellular fitness after *FN1* knockdown and subsequent treatment with imatinib (IM, 2 µM), nilotinib (N, 0.1 µM), dasatinib (D, 0.01 µM), bosutinib (B, 0.02 µM), ponatinib (P, 0.006 µM) and asciminib (A, 0.05 µM) measured on the level of **(C)** total cell numbers determined by trypan blue staining and **(D)** Ki-67 expression. Data were normalized to NC. Statistical analysis was performed using one-way ANOVA followed by Dunnett’s test or Student’s t-test. *N* = 3. Error bars indicate standard deviation. *p < 0.05, **p < 0.01, ***p < 0.001.

Since our previous study demonstrated that *FN1* downregulation decreased the sensitivity of K-562 cells to imatinib (Kaehler et al., 2022), we were interested whether similar effects could be observed with other BCR::ABL1 TKIs. SiRNA-mediated *FN1* knockdown in treatment-naïve K-562 cells resulted in a significant reduction of *FN1* mRNA expression (*p* = 0.001, Figure 2A) and a modest decrease in cell adhesion (*p* = 0.02, Figure 2B) compared to the negative control. Subsequently, cells were exposed to 2 µM imatinib (IM), 0.1 µM nilotinib (N), 0.01 µM dasatinib (D), 0.02 µM bosutinib (B), 0.006 µM ponatinib (P) or 0.05 µM asciminib (A). An increase in cell number was observed for all tested compounds (ranging from *N*: 25.3%, *p* = 0.04 to P: 53.0%, *p* = 0.02, Figure 2C), accompanied by significantly elevated proliferation rates (ranging between D: 9.2%, *p* = 0.03 to A: 346%, *p* < 0.001, Figure 2D; Supplementary Table 2). A comparable effect was detected following combined imatinib and nilotinib treatment, reflected by increased cell number and proliferation rates after *FN1* knockdown (Supplementary Figures S2C,D). Overall, these findings indicate that *FN1* downregulation is associated with reduced sensitivity to BCR::ABL1 TKI.

### 3.3 Restoration of *FN1* expression in TKI-resistant cells re-sensitizes the cells to TKI treatment

**FIGURE 3:**
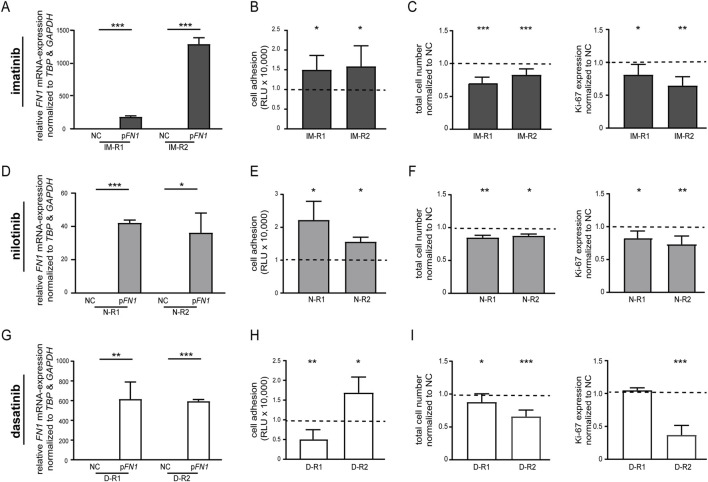
Rescue of *FN1* expression in TKI resistance restores TKI susceptibility. Restoration of *FN1* expression by transfection of a *FN1*-encoding plasmid (p*FN1*) in **(A–C)** imatinib-, **(D–F)** nilotinib- and **(G–I)** dasatinib-resistant cells. **(A,D,G)** *FN1* mRNA expression after transfection with FN1 or the empty vector control-transfected cells (NC) measured by RT-PCR and compared to *TBP* and *GAPDH*. **(B,E,H)** Cell adhesion analyzed by binding to Matrigel-coated surfaces. **(C,F,I)** Total cell numbers determined by trypan blue staining and Ki-67 expression after exposure to the respective TKI concentration (IM: 2 μM, N: 0.1 µM, D: 0.01 µM). Data were normalized to NC and analyzed using student’s t-tests. For each TKI, two biological replicate cell lines are shown (imatinib: IM-R1, IM-R2; nilotinib: N-R1, N-R2; dasatinib: D-R1, D-R2). *N* = 3. Error bars indicate standard deviation. *p < 0.05, **p < 0.01, ***p < 0.001.

Conversely, restoration of FN1 expression in imatinib-resistant K-562 through transfection with an *FN1*-encoding plasmid (Figure 3A) resulted in increased cell adhesion (IM-R1: 49.8%, *p* = 0.04; IM-R2: 50.0%, *p* = 0.04, Figure 3B; Supplementary Table 2). Decreases in cell numbers (IM-R1: −34.2%, *p* < 0.001, IM-R2: −18.9%, *p* < 0.001) and proliferation rate (IM-R1: −23.5%, *p* = 0.04, IM-R2: -40.5%, *p* = 0.003) indicated enhanced sensitivity to imatinib (Figure 3C; Supplementary Table 2). In nilotinib and dasatinib resistance, reduced cell numbers (nilotinib: R1: −27.1%, *p* = 0.003, R2: −14.6%, *p* = 0.04; dasatinib: R1: −13.3%, *p* = 0.02, R2: −34.0%, *p* < 0.001), decreased proliferation rates (nilotinib: R1: −16.3%, *p* = 0.02, R2: −26.9%, *p* = 0.002; dasatinib: R2: −63.0%, *p* < 0.001) were also observed following *FN1* restoration. In nilotinib resistance, an increased cell adhesion (nilotinib: R1: 111%, *p* = 0.03, R2: 50.5%, *p* = 0.04; Figures 3D–I; Supplementary Table 2) was detected, however, in dasatinib resistance, *FN1* transfection did not consistently alter cell adhesion (Figure 3H; Supplementary Table 2). In cells resistant to both imatinib and nilotinib, *FN1* restoration similarly re-sensitized the cells to TKI treatment, as reflected by reduced cell number and proliferation, together with significantly increased adhesion (Supplementary Figures S2E–G). These findings demonstrate that re-expression of *FN1* can counteract TKI resistance and partially re-sensitize the cells to BCR::ABL1 inhibition.

### 3.4 *FN1* re-expression overcomes TKI resistance independent from BCR::ABL1 kinase mutation

**FIGURE 4:**
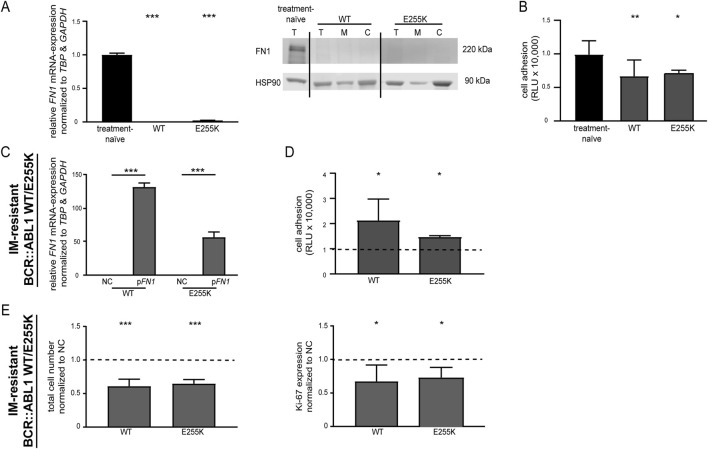
Restoration of *FN1* expression in TKI-resistant K-562 cells with acquired BCR::ABL1 p. E255K mutation. **(A,B)** FN1 expression and cell adhesion in TKI-resistant K-562 cells. **(A)** *FN1* mRNA expression and protein levels and **(B)** cell adhesion of E255K and BCR::ABL1 wild-type (WT) compared to treatment-naïve K-562 cells. **(C–E)** Rescue of *FN1* expression in p. E255K (E225K) and BCR::ABL1 WT cells by transfection of a *FN1*-encoding plasmid (p*FN1*) compared to empty vector control-transfected cells (NC). **(C)** *FN1* mRNA expression and **(D)** cell adhesion analyses after transfection, as well as **(E)** 2 µM imatinib treatment with subsequent analyses of total cell number and proliferation. *FN1* mRNA expression was measured by RT-PCR and compared to *TBP* and *GAPDH*. Protein levels were obtained by immunoblotting and compared to HSP90. Cell adhesion was measured by binding to Matrigel-coated surfaces. Data were normalized to NC. Statistical analysis was performed using one-way ANOVA followed by Dunnett’s test or Student’s t-test. *N* = 3. Error bars indicate standard deviation. *p < 0.05, **p < 0.01, ***p < 0.001. T, total protein; M, membrane fraction; **(C)**, cytosolic fraction.

In approximately 50% of cases, therapy resistance in CML is caused by mutations in the BCR::ABL1 kinase that prevent TKI binding. This raised the question of whether the effects of *FN1* can still be observed in the presence of BCR::ABL1 mutations. To investigate this, we compared cells from our in vitro-model carrying the acquired p. E255K mutation in ABL1 (NP:005148.2:p.Glu255Lys, rs121913448, COSV59235418) – a mutation associated with imatinib resistance–to imatinib-resistant cells without BCR::ABL1 mutations (wild-type (WT)). In cells harboring p. E255K, *FN1* expression was downregulated at both the mRNA and protein levels compared to treatment-naïve K-562 cells. A similar downregulation was observed in imatinib-resistant cells without BCR::ABL1 mutations (Figure 4A). Additionally, cell adhesion was reduced in both cell lines (WT: −40.6%, *p* = 0.005; E255K: −36.4%, *p* = 0.04, Figure 4B). Restoration of *FN1* expression in BCR::ABL1 p. E255K cells (*p* < 0.001) led to an increase in cell adhesion (40.5%, *p* = 0.04), and under imatinib exposure, resulted in a decreased cell number (−35.2%, *p* < 0.001) and proliferation (−28.7%, *p* = 0.03). Similar effects were observed in BCR::ABL1 wild-type imatinib-resistant cells (cell number: −39.1%, *p* < 0.001; proliferation: −32.7%, *p* = 0.04, Figures 4C–E). These data indicate that restoring *FN1* expression can re-sensitize cells to imatinib, even in the presence of BCR::ABL1 p. E255K mutation.

### 3.5 *FN1* improves the TKI response of cells overexpressing BCR::ABL1 wild-type or T315I

**FIGURE 5:**
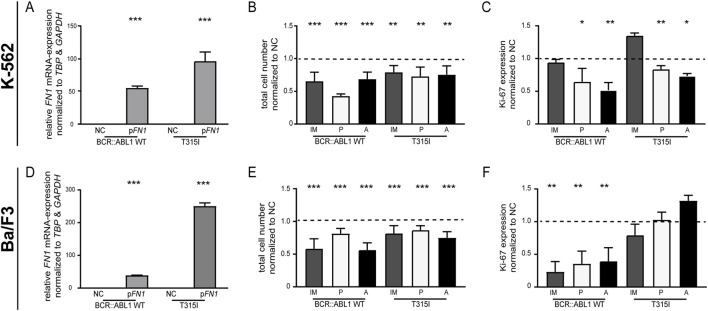
Role of *FN1* in K-562 and Ba/F3 cells overexpressing BCR::ABL1 wild-type or p. T315I. **(A–C)** K-562 cells harboring BCR::ABL1 wild-type (WT) or p. T315I (T315I) were transfected with a *FN1*-encoding plasmid (p*FN1*) or a negative control (NC) and subsequently exposed to the indicated TKIs. **(A)** *FN1* mRNA expression after transfection analyzed by RT-qPCR and normalized to *GAPDH* and *TBP*. **(B)** Total cell numbers determined by trypan blue staining and **(C)** proliferation analyzed by Ki-67 expression after exposure to 2 µM imatinib (IM), 0.006 µM ponatinib (P) or 0.05 µM asciminib **(A)**. **(D–F)** Ba/F3 cells harboring BCR::ABL1 WT or p. T315I were transfected with a *FN1*-encoding plasmid followed by TKI treatment. **(D)** *FN1* mRNA expression after transfection analyzed by RT-qPCR and normalized to *GAPDH* and *TBP*. **(E)** Total cell numbers and **(F)** proliferation after treatment with 2 µM imatinib (IM), 0.006 µM ponatinib (P) or 0.05 µM asciminib (A). Data were normalized to NC and analyzed using one-way ANOVA with subsequent Dunnett’s test or Student’s t-test. *N* = 3. Error bars indicate standard deviation. *p < 0.05, **p < 0.01, ***p < 0.001.

Among BCR::ABL1 mutations, the gatekeeper mutation p. T315I is particular critical for therapy failure in CML. This prompted us to investigate whether the influence of *FN1* could also be observed in the presence of this mutation. To this end, K-562 cells were stably transfected to overexpress either BCR::ABL1 wild-type or the p. Tyr315Ile (T315I) mutation, thereby introducing TKI resistance. Successful *FN1* overexpression was confirmed in both BCR::ABL1 WT (*p* < 0.001) and T315I cells (*p* < 0.001, Figure 5A). *FN1* overexpression did not uniformly affect cell adhesion: While an increase was observed in BCR::ABL1 WT cells (33.8%, *p* = 0.03), a decrease occurred in p. T315I cells (−47.3%, *p* < 0.001, Supplementary Figure S3A). Upon treatment with imatinib, ponatinib or asciminib, a significant reduction in the cell count was observed in both BCR::ABL1 WT (ranging asciminib: −38.2%, *p* < 0.001 to ponatinib: −59.9%, *p* < 0.001) and p. T315I cells (ranging from imatinib: −23.6%, *p* = 0.002 to ponatinib: −27.7%, *p* = 0.003; Figure 5B; Supplementary Table 2).

Similarly, cell proliferation was significantly reduced following treatment with ponatinib and asciminib in both cells lines (BCR::ABL1 WT: ponatinib: −40.4%, *p* = 0.01; ascitinib: −48.4%, *p* = 0.003; T315I: ponatinib: −19.3%, *p* = 0.009; ascitinib: −32.0%, *p* = 0.04), but not after treatment with imatinib (Figure 5C; Supplementary Table 2).

To exclude potential cell line-specific effects, Ba/F3 murine B cells being TKI-resistant by overexpression of BCR::ABL1 wild-type or the p. T315I mutation were transfected with FN1 and subsequently exposed to imatinib, ponatinib or asciminib. Following successful *FN1* overexpression (WT: *p* < 0.001; T315I: *p* < 0.001, Figure 5D) and TKI exposure, cell numbers were significantly reduced in both BCR::ABL1 WT (ranging from ponatinib: −18.7%, *p* < 0.001 to imatinib: −43.6%, *p* < 0.001) and p. T315I cells (ranging from ponatinib: −13.4%, *p* < 0.001 to ascitinib: −25.1%, *p* = 0.001, Figure 5E; Supplementary Table 2).

Similarly, proliferation was significantly decreased in BCR::ABL1 WT cells (ranging from ascitinib: −60.4%, *p* = 0.001 to imatinib: −76.8%, *p* = 0.009), but not in p. T315 cells (Figure 5E; Supplementary Table 2). In both cell lines, cell viability was also reduced following TKI treatment (Supplementary Figure S3B; Supplementary Table 2).

These findings suggest that restoration of FN1 can partially overcome TKI resistance, even in the presence of the BCR::ABL1 gatekeeper mutation p. T351I.

### 3.6 FN1 localization and pathway analysis after rescue of its expression in TKI-resistant cells

**FIGURE 6:**
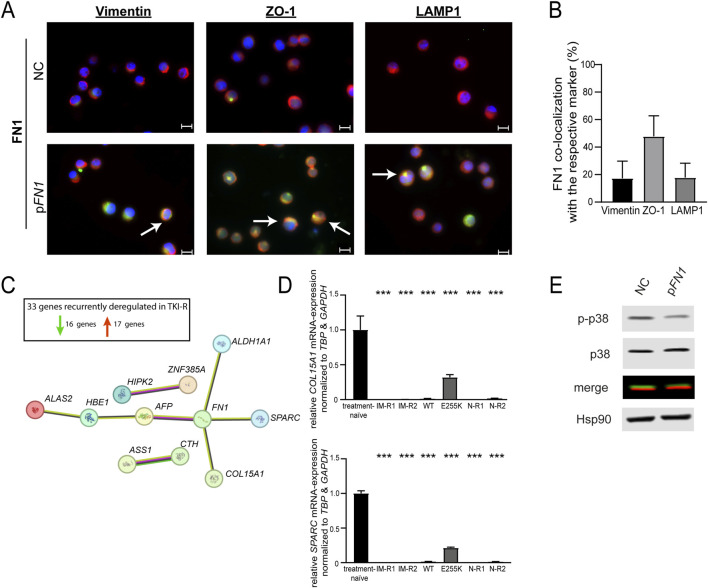
Localization and signaling pathway adaption after restoration of *FN1* expression in imatinib-resistant cells. **(A,B)** Immunofluorescence staining of imatinib-resistant K-562 cells transfected with the negative control (NC) or a *FN1*-encoding plasmid (p*FN1*). FN1 was stained using Alexa Fluor 488 (green) with co-staining of vimentin, ZO-1 or LAMP1 using Alexa Fluor 594 (red) and DAPI (blue). Depicted is one representative picture for each staining of *N* = 3 as merge picture. 63× magnification. Bar = 10 µm. Arrows indicate cytosolic, membrane and lysosomal staining. **(B)** FN1 co-localization with vimentin, ZO-1 or LAMP1 after densitometric analysis of *N* = 3 given in %. Error bars indicate standard deviation. **(C,D)** Recurrent genome-wide gene expression changes in TKI resistance. **(C)** STRING interaction network obtained for 33 recurrently deregulated genes in imatinib- and nilotinib-resistant cell lines compared to treatment-naïve cells. Pink: experimentally determined, green: gene neighborhood, yellow: textmining, black: co-expression. Red arrow indicates number of upregulated genes; green number of downregulated genes. **(D)** mRNA expression of *COL15A1* and *SPARC* analyzed by RT-qPCR. Expression was investigated in imatinib-resistant cells without *BCR::ABL1* mutations (IM-R1, IM-R2, WT), imatinib-resistant cells harboring BCR::ABL1 p. E255K (E255K), as well as nilotinib-resistant cell (N-R1, N-R2) compared to treatment-naïve K-562 cells. Data were normalized to *GAPDH* and *TBP* and treatment-naïve cells. Statistical analysis was performed using one-way ANOVA followed by Dunnett’s test. *N* = 3. Error bars indicate standard deviation. ***p < 0.001. **(E)** Phosphorylation of p-38 after restoration of *FN1* expression in nilotinib-resistant cells. HSP90 is shown as a housekeeping protein. Depicted is one representative blot out of *N* = 3. P-p38, phosho-p-38.

To investigate the subcellular localization of FN1 after rescue of *FN1* expression in TKI-resistant cells, FN1 was co-stained with vimentin (a cytoplasmatic marker), ZO-1 (a plasma membrane marker) and LAMP1 (a lysosomal marker) (Figure 6A; Supplementary Figure S4). An increase in green fluorescence indicated successful *FN1* restoration compared to negative control-transfected cells (Figures 6A,B). FN1 was primarily localized at the plasma membrane, as the shown by substantial co-localization with ZO-1. FN1 was hardly detected in lysosomes (as indicated by arrows) and showed minimal presence in the cytoplasm (Figures 6A,B). These findings suggest that, upon restoration of its expression, FN1 predominantly is localized at the plasma membrane in TKI-resistant cells.

The rescue of *FN1* expression in TKI-resistant cells led to a consistent improvement in TKI susceptibility thereby raising the question on the underlying mechanism. To identify potential mediators of the deregulated FN1 signaling in TKI resistance, genome-wide expression profiles of imatinib- and nilotinib-resistant cells were analyzed using previously published datasets (GSE227347 and GSE203342, (Kaehler et al., 2022)). Specifically, recurrent gene expression changes accompanying *FN1* downregulation were identified. By filtering for these genes in TKI-resistant cells, either imatinib resistant (harboring the BCR::ABL1 p. E255K mutation or wild-type) or nilotinib-resistant-33 genes were found with consistent expression changes across all six analyzed TKI-resistant sublines (Figure 6C). Of these, 16 genes were recurrently downregulated and 17 were upregulated compared to treatment-naïve cells.

Network analysis of these genes revealed direct interactions between FN1 and alpha-fetoprotein (*AFP*), aldehyde dehydrogenase 1 family member A1 (*ALDH1A1*), collagen type XV alpha-1 chain (*COL15A1*) and osteonectin/secreted protein acidic and rich in cysteine (*SPARC*) with *FN1* (Figure 6C). Subsequent validation of mRNA expression by RT-qPCR confirmed consistent downregulation of *COL15A1* (for all sublines: *p* < 0.001) and *SPARC* (for all sublines: *p* < 0.001) in TKI-resistant cells compared to treatment-naïve controls (Figure 6D).

Given that *SPARC* is involved in extracellular matrix formation, is functionally linked to FN1, and has previously been reported to be deregulated in CML (Nian et al., 2022), further transfection experiments were performed. Restoration of *SPARC* expression in imatinib-resistant cells led to a significant reduction in cell number (−25.5%, *p* = 0.02), although cell proliferation was not significantly altered compared to negative control-transfected cells under imatinib treatment (Supplementary Figure S5).

The FN1 (but also SPARC) signaling pathway involves an activation of p-38. In a previous study, we demonstrated that the rescue of *FN1* expression in imatinib-resistant cells resulted in a diminished phosphorylation of p-38 and ERK with the strongest reduction observed in phospho-p38 (Kaehler et al., 2022). To this end, p-38 activation in nilotinib-resistant cells was analyzed as a surrogate parameter. Following the rescue of *FN1*, a reduced phosphorylation of p-38 was detected in comparison to negative control-transfected cells thereby indicating a diminished proliferative signaling (Figure 6E). Taken together, these data suggest that the presence of FN1 results in diminished proliferative signaling, as indicated by reduced phosphorylation of p-38, potentially partially mediated by *SPARC*.

### 3.7 *FN1* expression is deregulated in CML patients

**FIGURE 7:**
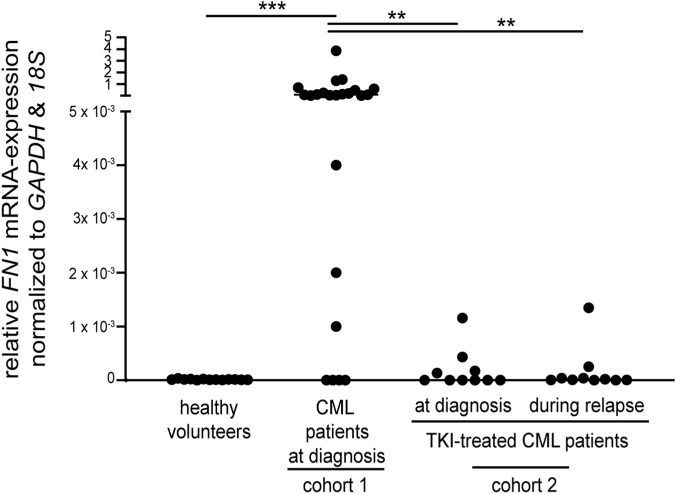
Expression pattern of *FN1* in CML. Samples were collected from 13 healthy volunteers and 33 CML patients: 23 CML patients at diagnosis (with unknown treatment outcome, cohort 1) and 10 TKI-treated CML patients (cohort 2) at diagnosis and suffering from relapse during TKI therapy (Supplementary Table 1). CML was diagnosed by FISH (Kaehler et al., 2021) or *BCR::ABL1* transcript evaluation. TKI relapse was stratified according to the *BCR::ABL1* transcript ≤0.1% (IS). *FN1* mRNA expression was measured by RT-qPCR and compared to *GAPDH* and *18S.* Samples with no *FN1* amplification were set to zero. Statistical analysis was performed using Mann-Whitney U tests. **p < 0.01, ***p < 0.001.

The in vitro-observation of downregulated *FN1* expression in TKI-resistant cell lines raised the question of whether these findings could also be confirmed in a clinical setting. To analyze this, peripheral blood samples were collected from 33 CML patients, including 23 CML patients at diagnosis (with unknown treatment outcome, cohort 1), 10 CML patients (cohort 2) at diagnosis, who later relapsed during TKI treatment, as well as 13 healthy volunteers. *FN1* mRNA expression was significantly increased in the peripheral blood from 23 CML patients at diagnosis (median [IQR 25–75]: 0.07 [0.002–0.47] compared to healthy volunteers (median [IQR 25–75]: 1.2 × 10^−5^ [7.1 × 10^−6^ – 3.5 × 10^−5^], *p* < 0.001, Figure 7). Furthermore, patients with relapsed CML showed a decrease in *FN1* levels in the peripheral blood at the time of diagnosis (median [IQR 25–75]: 2.1 × 10^−6^ [5.6 × 10^−7^ – 2.4 × 10^−4^], *p* = 0.003), as well as during the loss of MMR compared to the CML cohort 1 (median [IQR 25–75]: 1.4 × 10^−5^ [3.8 × 10^−6^ – 9.2 × 10^−5^], *p* = 0.003, Figure 7). Interestingly, there were seven patients in the CML cohort 1 with marginal *FN1* expression levels below 5 × 10^−3^.

## 4 Discussion

In this study, we initially confirmed the role of *FN1* in TKI-resistant CML cells demonstrating that diminished *FN1* expression was associated with TKI resistance. Furthermore, *FN1* knockdown in TKI-treatment-naïve cells impeded the sensitivity to all tested BCR::ABL1 TKIs, namely, imatinib, nilotinib, dasatinib, bosutinib, ponatinib and asciminib. Conversely, the restoration of *FN1* expression in TKI-resistant cells was sufficient to reestablish TKI sensitivity, even in the presence of BCR::ABL1 mutations. Furthermore, also in the clinical context, *FN1* expression was deregulated in leukocytes derived from CML patients.

FN1 is an ECM protein that plays a physiological role in cell adhesion and differentiation through its interaction with integrins (Pankov and Yamada, 2002). In the context of solid tumors, FN1 has been demonstrated to play a pivotal role in various hallmark features of cancer, including proliferation, migration, invasion, angiogenesis, metastasis and epithelial-mesenchymal transition (Wang and Hielscher, 2017; Lin et al., 2019). While the presence of FN1 in the ECM has been shown to promote tumor progression and formation of metastasis by activating pro-proliferative signals in certain cancers, it is paradoxically associated with a better prognosis (Lin et al., 2019; Farooq et al., 2023). However, studies examining its role in malignant progression in hematopoietic tumors remain limited. Cell adhesion to ECM components, particularly FN1, induced by integrins has been demonstrated to mediate drug resistance and anti-apoptotic signaling, known as cell adhesion-mediated drug resistance (Damiano et al., 2001). A multitude of studies have demonstrated that cell adhesion plays a pivotal role in leukemia, given its impact on the interaction of tumor cells with the bone marrow microenvironment or stroma (Krause and Scadden, 2015). Abnormalities of the cytoskeleton and increased cell adhesion to FN1 mediated by BCR::ABL1 have been observed in CML patients, while BCR::ABL1 mutations have been associated with reduced FN1 binding (Li et al., 2007). Nevertheless, the present study analyzed the role of FN1 itself in TKI resistance, rather than the binding to ECM molecules. This suggests that FN1, as part of the cell adhesion signaling, is involved in the development of TKI resistance. Our data demonstrate that not only the adhesion to ECM proteins can be altered in drug resistance, but also FN1 itself and its direct influence on TKI resistance play a role in this context.

Interestingly, *FN1* expression was recurrently downregulated in TKI-resistant K-562 cells, while no significant reduction was observed in imatinib-resistant LAMA-84 cells and it remained undetected in NALM-20 Ph + ALL cells. The K-562 cells were derived from a 53-year-old CML patient with CML in blast crisis, who had not received any treatment, while the LAMA-84 cells were derived from a 29-year-old patient with CML undergoing busulfan therapy (Lozzio and Lozzio, 1975; Seigneurin et al., 1987). Consequently, the observed variation in *FN1* expression between the two CML cell lines may be attributable to the distinct treatment modalities employed. Given that K-562 cells are CML blasts, they should be at least to some extent comparable to cells of an acute leukemia (Calabretta and Perrotti, 2004), e.g., B cell precursor leukemia Ph + ALL NALM-20 cells (Matsuo et al., 1991). However, *FN1* could not be detected in these cells. These disparities are presumably attributable to the distinct genetic backgrounds of the two cancer entities. Given that the leukemic stem cell niche in Ph + ALL exhibits distinct characteristics when compared to myeloid cancers, as, e.g., visible by expression of endothelial cell markers, such as VE-cadherine (Conforti et al., 2013), this potentially provides an explanation for the observed differences.

BCR::ABL1 overexpression and mutation, particularly p. T315I, have been identified as the cause of at least half of the cases of TKI resistance (Bixby and Talpaz, 2011; de Lavallade and Kizilors, 2016; Kaehler and Cascorbi, 2021). Gene mutations, but also other mechanisms of TKI resistance, necessitated the development of novel TKIs, in particular ponatinib and the allosteric inhibitor asciminib (Schoepfer et al., 2018; Luciano et al., 2020) being able to overcome these therapeutic limitations. Nevertheless, therapeutic options to circumvent the BCR::ABL1-mediated resistance apart from targeting of the kinase remain elusive. In the present study, *FN1* expression was also found to be downregulated in cells that intrinsically harbor the BCR::ABL1 mutation p. E255K, which is among the most prevalent BCR::ABL1 mutations (Majumdar et al., 2024). The restoration of *FN1* expression in these resistant cells likewise re-established TKI susceptibility. Furthermore, transfection of a *FN1*-encoding plasmid into K-562 and Ba/F3 cells, both of which are TKI-resistant due to the expression of the BCR::ABL1 wild-type or p. T315I, resulted in increased TKI sensitivity despite variable transfection efficiencies. Notably, this effect was observed not only for ponatinib or asciminib, but also during exposure to imatinib, which is incapable of inhibiting the BCR::ABL1 kinase in the presence of the p. T315I mutation. These findings suggest that FN1 is involved in a signaling pathway downstream of the BCR::ABL1 kinase leading to the inhibition of proliferative and anti-apoptotic signaling. This hypothesis is supported by a study on BCR::ABL1 p. T315I-positive CML in mice demonstrating that the presence of the p. T315I mutation affected cell adhesion and deposition of FN1 thereby enhancing malignant progression, while the administration of FN1 or an integrin-like kinase inhibitor to enhance FN1 expression increased the overall survival of CML cells (Kumar et al., 2020). These findings align with our observation of increased *FN1* expression in CML patients prior to treatment.

In TKI resistance, our findings indicated that FN1 restoration in TKI-resistant cell lines led to a partial overcoming of the resistance against the respective TKI. This phenomenon was accompanied by a reduction in proliferative signaling, as evidenced by a decrease in p-38 phosphorylation. This finding is also consistent with our previous research, which demonstrated that the restoration of FN1 in imatinib-resistant cells resulted in a reduction in p-38 and ERK phosphorylation, possibly mediated via the focal adhesion kinase (FAK) (Kaehler et al., 2022). This finding indicates that FN1-mediated cell adhesion signaling circumvents BCR::ABL1 inhibition suggesting that this is a promising target to overcome TKI resistance even in the presence of BCR::ABL1 mutations. In addition, we observed that *SPARC* is also recurrently downregulated alongside FN1. In a model of endometrial cancer, it was demonstrated that SPARC activates fibroblasts in the presence of FN1, which itself was secreted by SPARC-expressing endometrial cancer cells, leading to enhanced mobility and invasion (Yoshida et al., 2021). The FN1-SPARC-axis is of particular interest as *SPARC* expression has been shown to be reduced in CML patients, while it is increased in the serum of CML patients after imatinib treatment (Giallongo et al., 2013; Nian et al., 2022). The same study from Giallongo et al., it has been demonstrated that exogenous SPARC diminished the proliferation of K-562 synergistically with imatinib treatment, which aligns with our findings. To this end, further studies are necessary to elucidate the mechanisms of *FN1* and the potential involvement of *SPARC* in TKI resistance.

Cell adhesion to Matrigel-coated surfaces was not consistently altered in TKI-resistant cell lines. Moreover, the restoration of *FN1* expression in resistant cell lines did not uniformly increase the binding to Matrigel, as evidenced in dasatinib-resistance. It has been shown that CML progenitor cells exhibit a diminished capacity for adhesion to the stroma, that can be reversed by the administration of interferon-alpha or imatinib, indicating that TKI treatment directly influences cell adhesion (Li et al., 2007; Obr et al., 2014; Krause and Scadden, 2015; Minciacchi et al., 2021). Conversely, binding to the ECM prevents apoptosis induced by imatinib, DNA damaging agents or gamma-radiation mainly facilitated by integrin α5β1 (Damiano et al., 2001). In our model, TKI susceptibility was restored in resistant cells after *FN1* transfection independent of alterations in cell adhesion. Thus, FN1-mediated effects cannot be exclusively attributed to alterations in cell adhesion capacity, but may also be a result of signaling pathway activation. Furthermore, future studies should also entail the examination of the interaction between stromal cells and both TKI-sensitive and -resistant CML cells.

In the present study, *FN1* expression was found to be also increased in CML patients at the time of diagnosis in comparison to healthy volunteers. In contrast, patients who relapsed after an initial response to TKI treatment exhibited reduced expression levels. *FN1* has been demonstrated to be ubiquitously expressed in bone marrow cells (Wirth et al., 2020), so the observed deregulation of its expression in CML is particularly interesting. *FN1* expression was found to be reduced in certain TKI-non-responders at the time of diagnosis, and during relapse. This finding suggests a potential correlation between *FN1* expression and TKI resistance. The present study incorporated only 33 CML patients, 10 of whom experienced relapses during TKI therapy with variable sampling time-points. Thus, a larger confirmatory clinical study should be conducted. Furthermore, as potential differences in the sample composition might interfere with the expression pattern, subsequent studies should include analyses of CD34^+^ cells or FISH to extrapolate the tumor cell count. This approach could also facilitate the analysis of TKI responders. Furthermore, given the potential of age to influence *FN1* expression, age-matched cohorts should be analyzed in a future study. Overall, our data indicate that FN1 may serve as a promising predictor of TKI response.

The deregulation of *FN1* expression *in vitro* and *in vivo*, along with the demonstrated overcoming of resistance after restoration of *FN1* expression raises the question of whether FN1 can be used as a therapeutic target. There is indeed some evidence that the inhibition of FN1 may prevent the growth and metastasis of solid tumors in cases when FN1 is overexpressed (Wang and Hielscher, 2017). However, as *FN1* is downregulated in TKI-resistant CML and its expression or downstream signaling has to be restored to overcome TKI resistance, alternative approaches are required. Since the role of FN1 is context- and tumor-dependent, further studies are necessary to investigate the signaling pathways and FN1 as a pharmacological target.

## 5 Conclusion

Our data indicate that *FN1* deregulation is a recurrent phenomenon of TKI-resistant CML, both *in vitro* and in a clinical setting, as *FN1* mRNA was absent in TKI-resistant cell lines and dysregulated in peripheral blood cells from CML patients. Moreover, *FN1* knockdown reduced susceptibility to BCR::ABL1 inhibitors, whereas restoration of *FN1* expression in TKI-resistant cells overcame resistance independently of BCR::ABL1 mutations or its overexpression, suggesting a remarkable role beyond established resistance mechanisms. In summary, our findings identify FN1-mediated cell adhesion signaling as a potential therapeutic target to overcome TKI resistance in CML.

## Data Availability

The original contributions presented in the study are included in the article/Supplementary Material, further inquiries can be directed to the corresponding author.
