# Supplementary material for: Association of fibronectin 1 deregulation with tyrosine kinase inhibitor resistance in chronic myeloid leukemia
Source: Front Cell Dev Biol. 2025 Dec 19;13:1725857. doi: 10.3389/fcell.2025.1725857 (PMC12757412; doi:10.3389/fcell.2025.1725857)

Supplementary Tables

**Supplementary Table 1.** Clinical data from chronic myeloid leukemia patients lacking response to TKI treatment. Samples were collected at diagnosis and at routine checkups during the treatment. Data include BCR::ABL1 breakpoints, major molecular remission status, TKI and treatment duration at the time of sample collection. Therapy duration indicates the time between TKI treatment onset and sample collection. The major molecular remission (MMR) was defined by a BCR::ABL1 transcript ≤0.1 % (IS). In all patients, mutations in *BCR::ABL1* were not detected.

| **Case ID** | **Age** | **BCR-ABL1 breakpoint** | **Major molecular remission status** | **TKI at collection date** | **Therapy duration (months)** |
| --- | --- | --- | --- | --- | --- |
| 1 | 49 | M-bcr (e14-a2) |  | - |  |
|  |  | M-bcr (e14-a2) | No MMR | dasatinib | 29 |
| 2 | 91 | M-bcr (e14-a2) |  |  |  |
|  |  | M-bcr (e14-a2) | No MMR | imatinib | 3 |
| 3 | 53 | M-bcr (e14-a2) |  | - |  |
|  |  | M-bcr (e14-a2) | No MMR | imatinib | 5 |
| 4 | 83 | M-bcr (e14-a2) |  | - |  |
|  |  | M-bcr (e14-a2) | No MMR | dasatinib | 42 |
| 5 | 93 | M-bcr (e14-a2) |  |  |  |
|  |  | M-bcr (e14-a2) | No MMR | n.d. | 3 |
| 6 | 65 | M-bcr (e13-a2) |  | - |  |
|  |  | M-bcr (e13-a2) | No MMR | n.d. | 3 |
| 7 | 80 | M-bcr (e14-a2) |  | - |  |
|  |  | M-bcr (e14-a2) | No MMR | imatinib | 3 |
| 8 | 61 | M-bcr (e14-a2) |  | - |  |
|  |  | M-bcr (e14-a2) | No MMR | nilotinib | 2 |
| 9 | 33 | M-bcr (e13-a2) |  | - |  |
|  |  | M-bcr (e13-a2) | No MMR | dasatinib | 2 |
| 10 | 72 | M-bcr (e14-a2) |  | - |  |
|  |  | M-bcr (e14-a2) | No MMR | nilotinib | 3 |

MMR: major molecular remission, n.d.: no data on the therapy available, -: no therapy.

**Supplementary Table 2.** Fold changes and p-values from the experimental data included into the present study. IM: imatinib, N: nilotinib, D: dasatinib, B: bosutinib, P: ponatinib, A: asciminib, R: replicate, n.s: not significant, -: no data; WT: wild-type.

| **Fig. 1: *FN1* expression and cell adhesion properties of TKI-resistant cell lines.** | | | | | | | | | |
| --- | --- | --- | --- | --- | --- | --- | --- | --- | --- |
|  | **IM-R1** | **IM-R2** | **mean** | **N-R1** | **N-R2** | **mean** | **D-R1** | **D-R2** | **mean** |
| ***FN1* expression** | p < 0.001 | p < 0.001 | - | p < 0.001 | p < 0.001 | - | -99.8 %  p < 0.001 | -99.9 %  p < 0.001 | - |
| **cell adhesion** | n.s. | -60 %  p = 0.01 | n.s. | n.s. | 53 %  p = 0.04 | n.s. | -65.5 %  p = 0.006 | -61 %  p = 0.03 | -63.3 %  p = 0.002 |

| **Fig. 2: *FN1* knockdown in treatment-naive K-562 cells decreases susceptibility to BCR::ABL1 TKI treatment.** | | | | | | |
| --- | --- | --- | --- | --- | --- | --- |
|  | **IM** | **N** | **D** | **B** | **P** | **A** |
| **cell number** | 52.9 %  p = 0.03 | 25.3 %  p = 0.04 | 26.2 %  p = 0.01 | 30.9 %  p < 0.001 | 53 %  p = 0.02 | 40.8 %  p < 0.001 |
| **Ki-67 expression** | 44.9 %  p = 0.04 | 81.5 %  p = 0.04 | 9.2 %  p = 0.03 | 92.7 %  p < 0.001 | 12.3 %  p = 0.04 | 346.3 %  p < 0.001 |

| **Fig. 3: Rescue of *FN1* expression in TKI resistance restores TKI susceptibility.** | | | | | | | | | |
| --- | --- | --- | --- | --- | --- | --- | --- | --- | --- |
|  | **IM-R1** | **IM-R2** | **mean** | **N-R1** | **N-R2** | **mean** | **D-R1** | **D-R2** | **mean** |
| ***FN1* expression** | p < 0.001 | p < 0.001 | - | p < 0.001 | p = 0.03 | - | p = 0.004 | p < 0.001 | - |
| **cell adhesion** | 49.8 %  p = 0.04 | 59.0 %  p = 0.04 | 52.6 %  p = 0.001 | 111 %  p = 0.03 | 50.5 %  p = 0.04 | 76.1 %  p = 0.004 | -49.5 %  p = 0.001 | 75.8 %  p = 0.01 | n.s. |
| **cell number** | -34.2 %  p < 0.001 | -18.9 %  p < 0.001 | -26.5 %  p < 0.001 | -27.1 %  p = 0.003 | -14.6 %  p = 0.04 | -16.8 %  p = 0.003 | -13.3 %  p = 0.02 | -34 %  p < 0.001 | -25 %  p < 0.001 |
| **Ki-67 expression** | -23.5 %  p = 0.04 | -40.5 %  p = 0.003 | -32 %  p < 0.001 | -16.3 %  p = 0.02 | -26.9 %  p = 0.002 | -21.6 %  p < 0.001 | n.s. | -63 %  p < 0.001 | -31.7 %  p = 0.03 |

| **Fig. 4: Restoration of *FN1* expression in TKI-resistant K-562 cells with acquired BCR::ABL1 p.E274K mutation.** | | |
| --- | --- | --- |
|  | **BCR:ABL WT** | **E274K** |
| **Compared to treatment-naive cells:** |  |  |
| *FN1* expression | p < 0.001 | p < 0.001 |
| cell adhesion | -40.6 %  p = 0.005 | -36.4 %  p = 0.04 |
| **After transfection:** |  |  |
| *FN1* expression | p < 0.001 | p < 0.001 |
| cell adhesion | 112.1 %  p = 0.02 | 40.5 %  p = 0.04 |
| cell number | -39.1 %  p < 0.001 | -35.2 %  p < 0.001 |
| Ki-67 expression | -32.7 %  p = 0.04 | -28.7 %  p = 0.03 |

| **Fig. 5 & Supplementary Fig. S3: Role of *FN1* in K-562 and Ba/F3 cells with BCR::ABL1 wild-type or p.T315I.** | | | | | | |
| --- | --- | --- | --- | --- | --- | --- |
| **K-562** | **BCR::ABL WT** | | | **T315I** | | |
| *FN1* expression | p < 0.001 | | | p < 0.001 | | |
| cell adhesion | 33.8 %  p = 0.03 | | | -47.3 %  p < 0.001 | | |
|  | **IM** | **P** | **A** | **IM** | **P** | **A** |
| cell number | -40.7 %  p < 0.001 | -59.9 %  p < 0.001 | -38.2 %  p < 0.001 | -23.6 %  p = 0.002 | -27.7 %  p = 0.003 | -26.9 %  p = 0.001 |
| Ki-67 expression | n.s. | -40.4 %  p = 0.01 | -48.4 %  p = 0.003 | n.s. | -19.3 %  p = 0.009 | -32 %  p = 0.04 |
| **Ba/F3** | **BCR::ABL WT** | | | **T315I** | | |
| *FN1* expression | p < 0.001 | | | p < 0.001 | | |
|  | **IM** | **P** | **A** | **IM** | **P** | **A** |
| cell number | -43.6 %  p < 0.001 | -18.7 %  p < 0.001 | -41.0 %  p < 0.001 | -19.8 %  p < 0.001 | -13.4 %  p < 0.001 | -25.1 %  p = 0.001 |
| Ki-67 expression | -76.8 %  p = 0.009 | -64.5 %  p = 0.002 | -60.4 %  p = 0.001 | n.s. | n.s. | n.s. |
| cell viability | -13.5 %  p = 0.02 | -26.9 %  p < 0.001 | -21.9 %  p = 0.006 | -9.1 %  p = 0.01 | -5.3 %  p = 0.04 | -12.7 %  p = 0.004 |

Supplementary Figures

**Supplementary Fig. S1. *FN1* expression in TKI-resistant LAMA-84 and NALM-20 cells.** *FN1* mRNA expression was measured using RT-qPCR in treatment-naïve and TKI-resistant LAMA-84 CML cells (resistant against 0.5 and 2 µM imatinib) and NALM-20 Ph+ ALL cells (resistant against 0.1 µM imatinib). Data were normalized to *TBP* and *GAPDH* and the respective treatment-naïve cells. In NALM-20 cells, *FN1* mRNA expression was below the detection threshold. Statistical analysis was performed using one-way ANOVA followed by Dunnett’s test or student’s t-test. N = 3. Error bars indicate standard deviation. IM: imatinib.


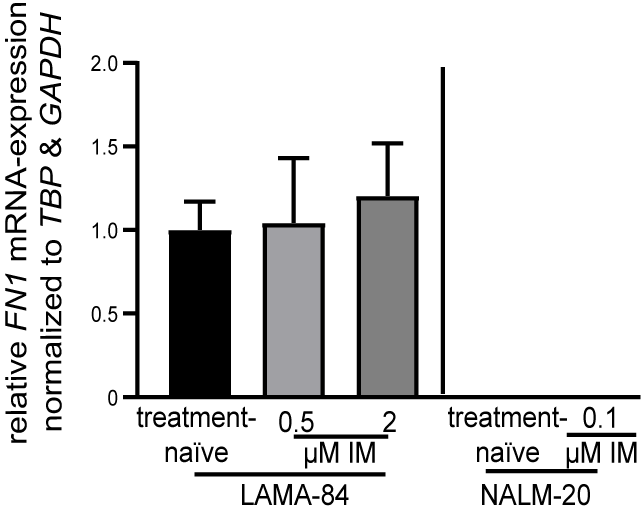


**Supplementary Fig. S2. Role of *FN1* in CML cells resistant against imatinib and nilotinib.** (A) *FN1* mRNA expression of CML cells resistant against imatinib (IM, 2 µM) and nilotinib (N, 0.1 µM) measured using RT-qPCR and normalized to *TBP*, *GAPDH* and treatment-naïve cells. (B) Cell adhesion of dual resistant cell lines normalized to native K‑562 cells measured by binding to Matrigel-coated surfaces. (C-D) SiRNA-mediated knockdown of *FN1* in treatment-naïve K-562 cells with subsequent combinatory treatment with IM and N. (C) Total cell numbers were obtained by trypan blue staining; (D) proliferation was analyzed by Ki-67 expression and compared to negative control-transfected cells (NC). (E-G) Restoration of *FN1* expression in cells resistant against imatinib and nilotinib. Rescue of *FN1* expression was performed by transfection of a *FN1*-encoding plasmid with subsequent analyses of (E) total cell numbers, (F) Ki-67 expression and (G) cell adhesion to Matrigel-coated surfaces. Data were normalized to negative control-transfected cells (NC). Statistical analysis was performed using one-way ANOVA followed by Dunnett’s test or student’s t-tests. Two cell lines were used, one of them being initially resistant to imatinib and subsequently nilotinib, and vice versa. N = 3. Error bars indicate standard deviation. *: p < 0.05, **: p < 0.01, ***: p < 0.001.


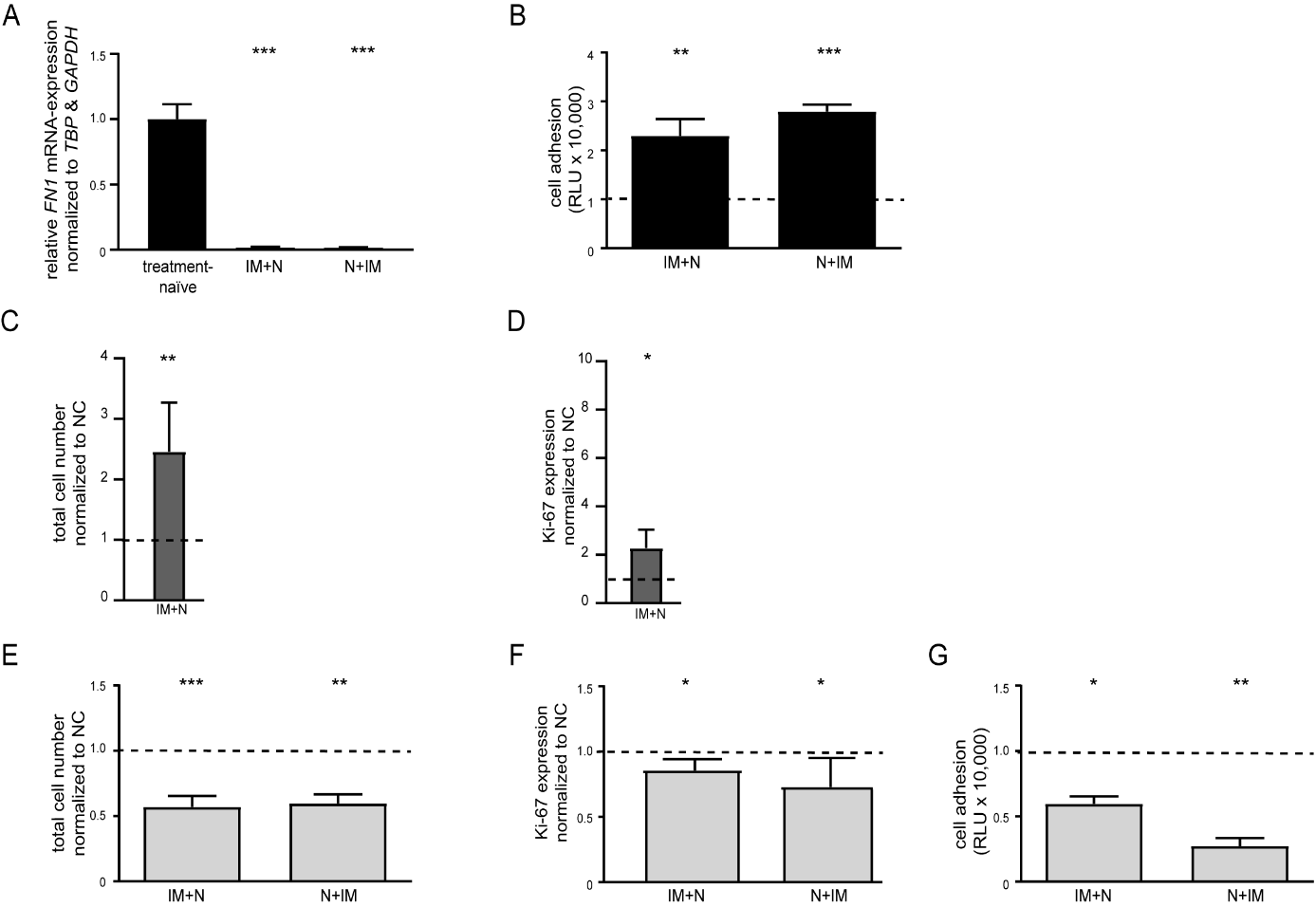


**Supplementary Fig. S3. *FN1*-transfection into K-562 and Ba/F3 cells overexpressing BCR::ABL1 wild-type or p.T315I mutation.** (A) Cell adhesion to Matrigel-coated surfaces of BCR::ABL1 wild-type (WT) or p.T315-expressing (T315I) K-562 cells after transfection of *FN1*. (B) Cell viability of BCR::ABL1 WT or p.T315I-expressing Ba/F3 cells after *FN1*-transfection. N = 3. Data normalized to empty vector-control transfection (NC). Statistical analysis was performed using student’s t-tests. Error bars indicate standard deviation. *: p < 0.05, **: p < 0.01, ***: p < 0.001. IM: imatinib, P: ponatinib, A: asciminib.

**
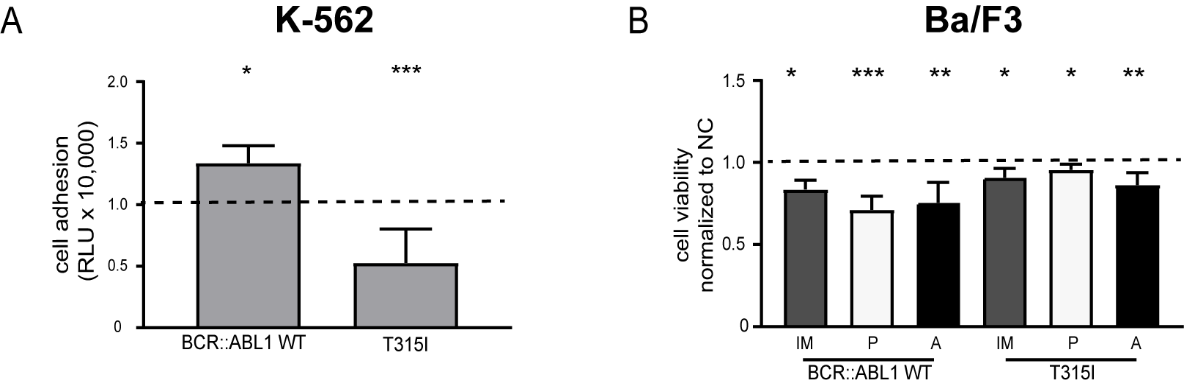
**

**Supplementary Fig. S4. Immunofluorescence staining of K-562 cells.** (A) Control staining for anti-mouse/Alexa Fluor 488, anti-rabbit/Alexa Fluor 594 and DAPI in samples without primary antibodies. (B-C) Immunofluorescence staining depicted as merge pictures, as well as the single channels in grey. Imatinib-resistant K‑562 cells were transfected with either the (B) negative control (NC) and (C) a *FN1*-encoding plasmid and stained for FN1 using Alexa Fluor 488 (green) and vimentin, ZO-1 or LAMP1 using Alexa Fluor 594 (red), as well as DAPI (blue). Depicted is one representative picture for each staining of N = 3. 63x magnification. Bar = 10 µm. Arrows indicate cytosolic, membrane and lysosomal staining.


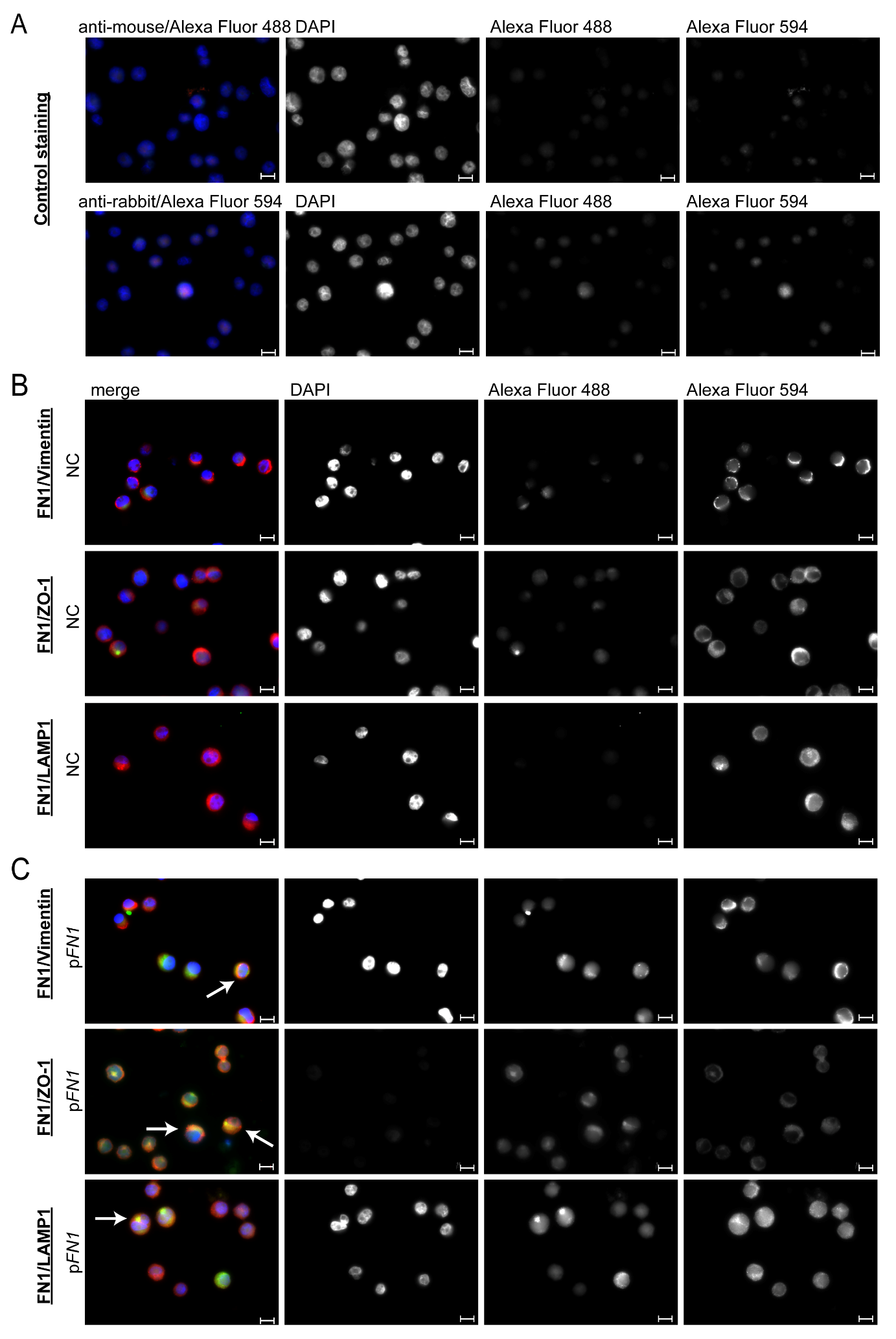


**Supplementary Fig. S5. Role of *SPARC* in imatinib resistance.** Imatinib-resistant K-562 cells were transfected with a *SPARC*-encoding plasmid (p*SPARC*) and cell fitness was subsequently analyzed. (A) mRNA expression after restoration of *SPARC* expression analyzed using RT-qPCR and normalized to *TBP* and *GAPDH*. (B) Cell numbers and (C) Ki-67 expression in the presence of imatinib after *SPARC* rescue in imatinib-resistant cells normalized to empty vector-control transfection (NC). N = 3. Statistical analysis was performed using student’s t-tests. Error bars indicate standard deviation. *: p < 0.05, ***: p < 0.001.


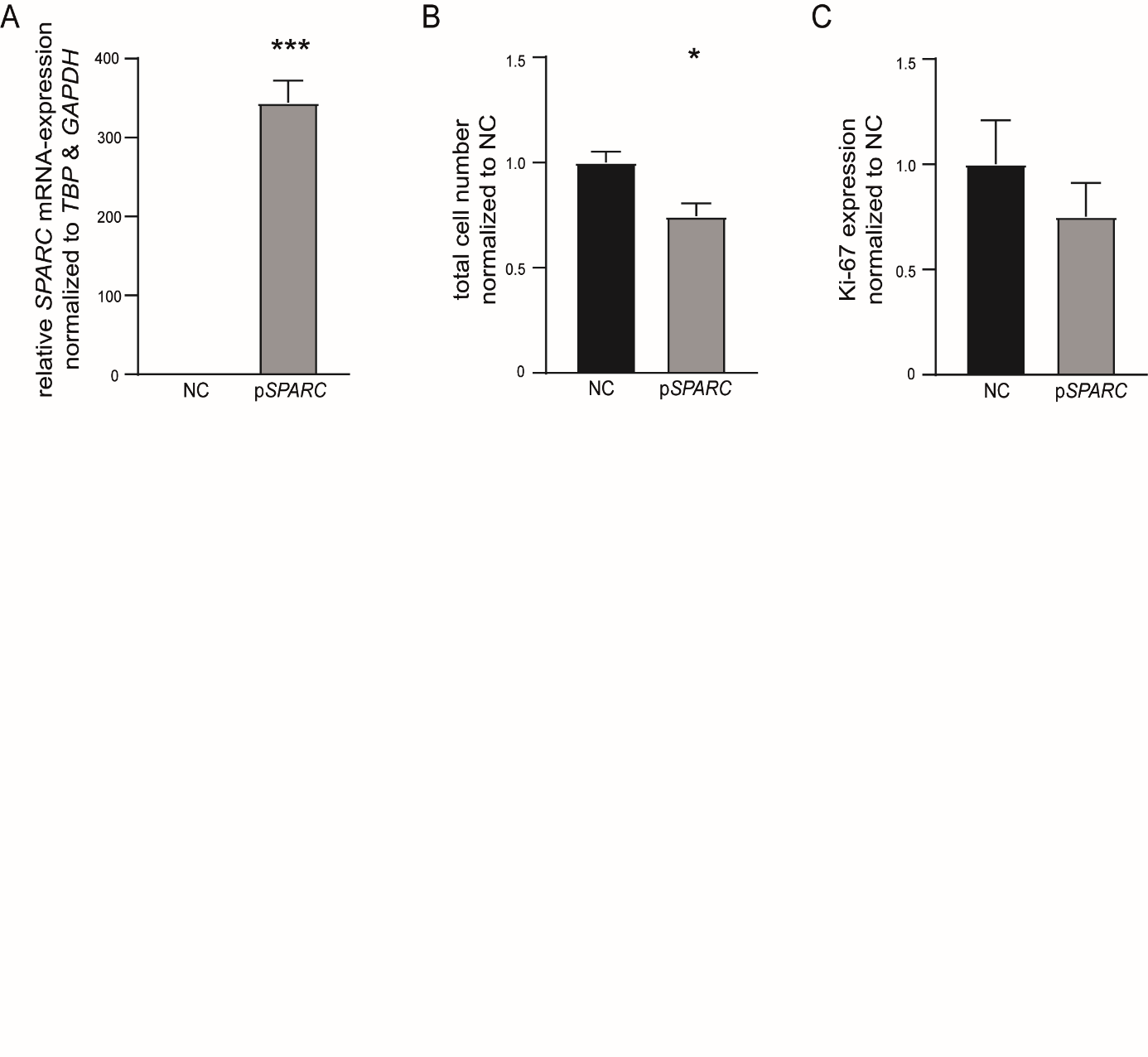

Supplement: Supplementary file 1 [file DataSheet1.docx]
